# How Sweet It Is: A Perspective on the Potential Anti-Tumor Role for SGLT2 Inhibitors

**DOI:** 10.34067/KID.0000000000000219

**Published:** 2023-09-28

**Authors:** Deepthi Gunasekaran, Anushree C. Shirali

**Affiliations:** Section of Nephrology, Yale University School of Medicine, New Haven, Connecticut

**Keywords:** SGLT2 inhibitors, acute interstitial nephritis, immune checkpoint inhibitors, cancer

The US Food and Drug Administration approved sodium-glucose cotransporter (SGLT) 2 inhibitors (SGLT2is) in 2013 for glucose lowering in patients with type II diabetes. Since then, these drugs have shown far-reaching effects beyond diabetes management. The (Empagliflozin) Cardiovascular Outcome Event Trial in Type 2 Diabetes Mellitus Patients trial sought to prove the cardiac safety of SGLT2is but went further to demonstrate that empagliflozin significantly reduced major adverse cardiac events, cardiovascular deaths, risk of heart failure hospitalization, and all-cause deaths.^1^ Several large clinical trials later confirmed these results in patients with heart failure with reduced as well as preserved ejection fraction and in patients with coronary artery disease.^2^ When exploratory findings from these trials hinted at improved kidney outcomes with SGLT2is, trials evaluating kidney-specific end points were conducted. Among these, the Canagliflozin and Renal Events in Diabetes with Established Nephropathy Clinical Evaluation trial^3^ was terminated early after showing a 30% lower risk of developing ESKD, doubling of creatinine or death from renal or cardiovascular causes. The Dapagliflozin and Prevention of Adverse Outcomes in CKD^4^ and Study of Heart and Kidney Protection with Empagliflozin^5^ trials demonstrated the benefit of SGLT2is even with advanced CKD, regardless of diabetes status or proteinuria. Collectively, these data suggest that SGLT2is possess pleiotropic mechanisms beyond glucosuria for end-organ protection, with manageable side effects, including polyuria and, less commonly, recurrent urinary tract infections and euglycemic ketoacidosis. Early trial data with dapagliflozin and empagliflozin had also reported a trend toward higher breast cancer and bladder cancer rates, respectively. However, murine studies found no tumor promoter or progressor potential for dapaglafozin.^6^ In addition, large meta-analyses consistently showed no increase in the overall risk of cancer or of specific cancer types.^6^

Emerging evidence now suggests that SGLTs may actually play a vital role in tumor cell homeostasis, raising the attractive prospect that SGLT2is may possess anticancer properties.^6,7^ The link between cancer growth and glucose dates to the pioneering studies by Otto Warburg describing increased glucose uptake and preferential use of glycolysis for energy in tumor cells versus normal cells.^6^ Glucose enters tumor cells using facilitative glucose transporters and SGLTs, such as SGLT1 and SLGT2.^6^ SGLTs belong to the large solute carrier family 5A and facilitate secondary active transport of glucose using the electrochemical Na^+^ gradient generated by Na^+^/K^+^-ATPase.^7^ SGLT1 is expressed in enterocytes lining small intestinal villi and is responsible for glucose absorption from the gut.^7^ SGLT2 is localized to the apical membrane of the S1 and S2 segments of proximal tubule epithelia and reabsorbs approximately 90% of filtered glucose.^7^ SGLT1 expression in the kidney is limited to the apical membrane of the S3 segment of the proximal tubule, where it reabsorbs the remaining filtered glucose.^7^ Specific to cancer cells, both SGLT1 and SGLT2 are upregulated in various cancer cells, including the lung, breast, liver, brain, and pancreas, and SGLT2 inhibition has shown reduced tumor growth in murine models.^6,7^ While glucose provision to cancer cells is believed to be the main driver for SGLT2-mediated tumor growth, SGLT2is are postulated to have several mechanisms of antineoplastic activity, including disruption of DNA/RNA synthesis and inhibition of cell cycle signaling pathways, such as mammalian target of rapamycin.^6^

**Figure 1.**
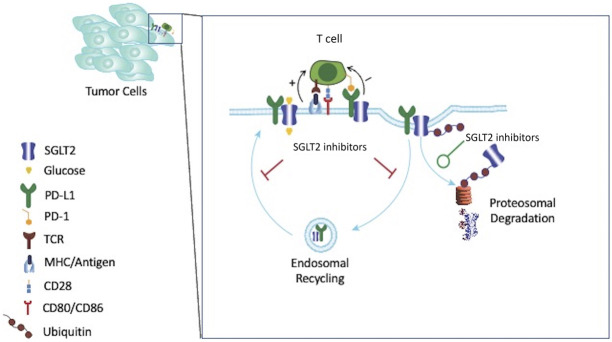
**A schematic representation of the interaction between SGLT2 and PD-L1 on cancer cells.** Tumor cells display antigens in an MHC-restricted fashion to T cells with an antigen-specific TCR. When costimulatory molecules (*e.g.*, CD28) on the T cells bind to their cognate receptor on tumor cells, T cells are activated. This process is negatively regulated by binding of inhibitory coreceptors, such as PD-1/PD-L1. PD-L1 is expressed on tumor cells and antigen-presenting cells. As with all cell-surface proteins, PD-L1 undergoes endocytosis to face either endosomal recycling back to the cell membrane or intracellular degradation. A recent study^8^ proposes that cell-surface SGLT2 colocalizes with PD-L1 to prevent its degradation by promoting endosomal recycling. Use of SGLT2 inhibitors discourages endosomal recycling (red lines) and promotes (green line) proteosomal degradation of PD-L1. MHC, major histocompatibility complex; PD-1, programmed death protein-1; PD-L1, programmed death protein ligand 1; SGLT2, sodium glucose cotransporter 2.

Intriguingly, a new study by Ding and colleagues offers the novel finding that SGLT2 may promote tumor growth through increased expression of the inhibitory immunoreceptor programmed death protein ligand 1 (PD-L1).^8^ In looking for novel PD-L1 inhibitors, they screened 98 clinically approved small molecule drugs on a patient-derived non–small cell lung cancer cell line with high PD-L1 expression. Canagliflozin was the most potent inhibitor of PD-L1 expression without affecting other immune checkpoint molecules. Use of small interfering RNA targeting SGLT2 abrogated the canagliflozin-induced inhibition of PD-L1, suggesting that this was a SGLT2-specific effect. Confocal microscopy showed that PD-L1 and SGLT2 colocalized on the cell surface through binding of their intracellular domains, a process which was disrupted by canagliflozin only if the sodium-binding site of canagliflozin on SGLT2 was not mutated, suggesting another on-target effect of SGLT2 inhibition. The authors next demonstrated that canagliflozin reduced stability of PD-L1 without affecting the mRNA expression of PD-L1. To mechanistically study the decreased half-life of cell-surface PD-L1 protein with SGLT2 inhibition, Ding and colleagues used an endocytosis inhibitor to inhibit internalization of cell-surface PD-L1 and found that this prevented canagliflozin-induced PD-L1 degradation. Internalized cell-surface proteins can either be trafficked back to the cell membrane or undergo lysosomal, autophagosomal, or proteasomal degradation. Thus, they next showed that use of a recycling endosome inhibitor or proteosome inhibitor, but not a lysosome inhibitor, impeded canagliflozin-mediated PD-L1 degradation. Taken together, these data suggest that canagliflozin inhibits endocytic recycling of PD-L1 and induces ubiquitination-mediated degradation of PD-L1 (Figure 1). Using two separate murine cancer models, they showed in vivo efficacy of canagliflozin to reduce tumor growth. Interestingly, in the CT26 immunocompetent mouse model, cotreatment of canagliflozin and anti–cytotoxic T-lymphocyte antigen (CTLA-4) antibody significantly reduced the size of tumors compared with either canagliflozin or anti–CTLA-4 monotherapy. The combination of anti–PD-L1 and anti–CTLA4 antibodies achieved a comparable therapeutic efficacy compared with the anti–CTLA4+canagliflozin group. Finally, the authors also looked at expression of SGLT2 on human non–small cell lung cancer biopsy specimens from patients treated with programmed death protein-1 (PD-1) and found increased progression-free and overall survival in patients whose biopsies displayed >50% SGLT2 expression, suggesting that SGLT2 expression may predict clinical response to immunotherapy.

Ding and colleagues have described a novel mechanism for antitumor effects of SGLT2 inhibitors, but several caveats remain. First, these data need to be replicated with other SGLT2is to elucidate whether the findings from the present study are a class effect. Second, most of the data presented are preclinical, and their applicability to patients remains to be seen. Third, there is presently no clear evidence that use of SGLT2is in patients with cancer leads to improvement in anticancer outcomes, although clinical trials are now beginning to use SGLT2is in conjunction with other established antineoplastic agents.^9,10^ Finally, if monoclonal antibodies against immune checkpoints, such as PD-L1, already exist, what would be the advantage of SGLT2 inhibition? By targeting inhibitory receptors, such as PD-1 or CTLA-4 on T cells as well as PD-L1 on tumor cells, immune checkpoint inhibitors (ICPIs) restore anticancer T-cell immunity. This has changed the landscape of modern oncology practice, but their use is complicated by off-target immune-related adverse events (irAEs), including kidney irAEs, such as AKI. Acute tubulointerstitial nephritis (ATIN) is the most common kidney biopsy-proven finding in ICPI-AKI, resulting in treatment disruption and corticosteroid administration.^11^ ICPI-AKI may lead to residual CKD and may recur on ICPI rechallenge.^11^ Thus, the pivotal question for nephrologists is whether SGLT2-mediated inhibition of immune checkpoints would lead to ATIN as seen with ICPIs. Despite its widespread use at present, there are only two case reports of ATIN associated with use of SGLT2is for standard indications.^12,13^ Assuming that the PD-L1/SGLT2 colocalization on tumor cells is present in vivo in humans, one potential explanation for this may be that the interaction between PD-L1 and SGLT2 as observed by Ding and colleagues is unique to cancer cells. Indeed, human kidney biopsy specimens show low levels of basal PD-L1 expression in a normal proximal tubular epithelium.^14^ Another explanation may be that PD-L1 inhibition is not as potent in inducing irAEs as PD-1 inhibition because the latter also affects the PD-L2 axis, which has its own immunoregulatory properties. Finally, current doses of SGLT2is used in glycemic, congestive heart failure, or CKD management may be insufficient to inhibit PD-L1. It is unclear whether higher doses of SGLT2is will be required for any anticancer benefit, and if so, whether such doses would be sufficient to inhibit PD-L1 expression and lead to irAEs. Separate from this, starting SGLT2is in any patient may transiently lower eGFR because of a diuretic effect. This may be particularly important in patients with cancer, especially those enrolled in clinical trials, in whom rising creatinine may trigger delays in anticancer therapy. Despite these unanswered questions or concerns, there is reason for cautious optimism. SGLT2 inhibitors are well-tolerated drugs which now have approved indications for use beyond their original intent. They have bridged the world of nephrology with cardiology and endocrinology, and they may do so yet again with oncology, potentially providing patients with cancer with additional therapeutic options and fewer kidney-related toxicities.
